# Valorization of Corn Steep Liquor and Glycerol for Fungal Chitosan Production by Mucorales from Brazilian Biomes: Structural Characterization and Antimicrobial Activity

**DOI:** 10.3390/molecules31162807

**Published:** 2026-08-12

**Authors:** Lúcia Raquel Ramos Berger, Thayza Christina Montenegro Stamford, Marcos Antonio B. de Lima, Danielle Silva Araújo, Mateus Henrique Freire Farias, Krause Gonçalves Silveira Albuquerque, Leonie Asfora Sarubbo, Mayri Alejandra Diaz De Rienzo, André Luiz Cabral Monteiro de Azevedo Santiago, Gerla Castello Branco Chinelate

**Affiliations:** 1 Departamento de Laboratórios Multiusuários, Campus Universitário Sede, Universidade Federal do Agreste de Pernambuco, Av. Bom Pastor, s/n° Boa Vista, Garanhuns 55292-270, Pernambuco, Brazil; luciaraquelramosberger@gmail.com (L.R.R.B.); gerla.chinelate@ufape.edu.br (G.C.B.C.); 2 School of Science, Engineering and Environment, University of Salford, Peel Building, Manchester M5 4WT, UK; m.a.diazderienzo@salford.ac.uk; 3 Laboratório de Microbiologia Aplicada-LaMAp, Centro de Ciências Médicas, Universidade Federal de Pernambuco, Av. da Engenharias, 317, Cidade Universitária, Recife 50670-420, Pernambuco, Brazil; 4 Departamento de Medicina Tropical, Centro de Ciências Médicas, Universidade Federal de Pernambuco, Av. da Engenharia, 531-611, Cidade Universitária, Recife 50730-120, Pernambuco, Brazil; 5 Programa de Pós-Graduação em Nutrição-PPGN, Universidade Federal de Pernambuco, Av. Prof. Moraes Rego, n° 1235, Cidade Universitária, Recife 50670-901, Pernambuco, Brazil; danielle.silvaa@ufpe.br; 6 Departamento de Biologia, Universidade Federal Rural de Pernambuco, Av. Dom Manoel de Medeiros, s/n°, Dois Irmãos, Recife 52171-900, Pernambuco, Brazil; marcos.barbosalima@ufrpe.br (M.A.B.d.L.); mateus.freire@ufrpe.br (M.H.F.F.); 7 Departamento de Micologia, Centro de Biociências, Universidade Federal de Pernambuco, Av. Prof. Moraes Rego, s/n, Cidade Universitária, Recife 50760-420, Pernambuco, Brazil; krause.albuquerque@ufpe.br (K.G.S.A.); andre.masantiago@ufpe.br (A.L.C.M.d.A.S.); 8 Instituto Avançado de Tecnologia e Inovação (IATI), Rua Potyra, n. 31, Prado, Recife 50070-280, Pernambuco, Brazil; leonie.sarubbo@unicap.br; 9 Escola de Tecnologia e Comunicação, Universidade Católica de Pernambuco (UNICAP), Rua do Príncipe, n. 526, Boa Vista, Recife 50050-900, Pernambuco, Brazil

**Keywords:** antimicrobial activity, circular economy, corn steep liquor, eco-friendly by-products, fungal chitosan, glycerol, Mucorales fungi

## Abstract

The sustainable reuse of industrial by-products in fermentation processes supports the transition toward a circular bioeconomy. This study evaluated the production yield of fungal chitosan (FuCho) by nine Mucorales strains, including novel species isolated from Brazilian biomes, cultivated in alternative culture media composed of corn steep liquor (CSL) and biodiesel-derived glycerol (GLY). Furthermore, the physicochemical properties, potential of irritation, and broad-spectrum antimicrobial activity of the extracted FuCho were investigated. Among the strains tested in CSL-GLY medium, FuCho yields ranged from 17.88 mg/g (*Absidia aguabelensis*) to 126.56 mg/g (*Absidia caatinguensis*). Optimization using a $2^{2}$ central composite rotatable design (CCRD) yielded maximum FuCho productions of 128.26 mg/g for *A. caatinguensis* (center point condition) and 164.92 mg/g for *Cunninghamella elegans* (lower CSL concentration). Physicochemical characterization revealed degrees of deacetylation of 85% (*A. caatinguensis*) and 75% (*C. elegans*), crystallinity indices of 36.74% and 37.93%, and viscometric molecular weights ($M_{v}$) of $1.6\times 10^{3}\;g/\mathrm{mol}$ and $2.0\times 10^{3}\;g/\mathrm{mol}$, respectively. In the HET-CAM assay, both FuCho samples were classified as non-irritating, exhibiting no inflammatory, vascular, or vasoconstrictive effects. FuCho demonstrated minimum inhibitory concentrations (MIC) against all tested pathogenic bacterial strains and *Candida* species, with *A. caatinguensis* FuCho displaying lower MIC values for the majority of microorganisms compared to *C. elegans* FuCho. Confocal laser scanning microscopy confirmed a significant reduction in microbial cell viability, driven by membrane disruption in *Escherichia coli* and *Staphylococcus aureus*. These findings validate the CSL-GLY mixture as a cost-effective, eco-friendly culture medium for producing high-value biopolymers with pharmaceutical potential, underscoring the biotechnological relevance of Brazilian Mucorales strains.

## 1. Introduction

Over the last few decades, natural polymers have been extensively investigated as eco-friendly alternatives to synthetic materials in the fields of medicine, pharmacy, cosmetics, agriculture, and food science [1]. Among these, fungal chitosan (FuCho)—a non-toxic, biocompatible, and biodegradable heteropolysaccharide derived from the deacetylation of chitin in the cell walls of Mucorales fungi—has emerged as a promising biopolymer [2]. Its unique polyelectrolytic properties, resulting from the protonation of free amino groups in acidic media, confer broad-spectrum antimicrobial activity that is particularly attractive for pharmaceutical and food safety applications [3,4].

However, the economic viability of FuCho production is often hampered by the high costs of traditional, chemically defined fermentation media [5]. To overcome this limitation, this work adopts a circular bioeconomy approach by valorizing agro-industrial and biodiesel by-products as low-cost, nutrient-rich substrates offers a sustainable alternative to the linear “take-make-dispose” model [6]. Two specific by-products, crude glycerol (GLY) and corn steep liquor (CSL), stand out as particularly promising nutrient sources for fungal cultivation and FuCho production.

Crude glycerol, an abundant residue generated during biodiesel production (approximately 1 kg of GLY per 9 kg of biodiesel), represents an important value-added feed material [7]. Its direct, unpurified utilization as a carbon source in fermentation provides an efficient pathway for biopolymer synthesis while simultaneously mitigating environmental impacts and reducing the carbon footprint of the biodiesel industry [7,8]. Similarly, corn steep liquor, a nutrient-rich effluent from corn wet milling, serves as a highly cost-effective source of organic nitrogen, amino acids, vitamins, and trace minerals [9,10]. Together, GLY (carbon source) and CSL (nitrogen and mineral source) offer complementary inputs, providing a practical, sustainable alternative to conventional media.

The order Mucorales comprises over 350 fungal species, predominantly saprobes, although some are parasites of animals, other fungi, and plants [11]. As metabolically versatile organisms capable of assimilating diverse carbon sources, these fungi adapt to distinct ecosystems by producing numerous secondary metabolites. Because the production of metabolites can vary according to each species and it is intrinsically influenced by environmental conditions, these strains isolated from unique Brazilian ecosystems may harbor untapped biotechnological potential.

Although more than 30 novel Mucorales species have been discovered in different Brazilian biomes since 2012 [12], no prior studies have evaluated these newly described species for FuCho or biopolymer production. Furthermore, there has been no research to evaluate the FuCho production by new species of Mucorales namely *Absidia caatinguensis*, *A. aguabelensis*, *Backusella constricta*, *Lichtheimia brasiliensis*, and *Mucor pernambucoensis* isolated from Caatinga and Brazilian Atlantic Rainforest biomes using a combination of GLY and CSL culture medium.

Therefore, this work aimed to: (i) establish the use of CSL and GLY as low-cost, circular bioeconomy-inspired substrates for FuCho production by Mucorales strains; and (ii) comprehensively evaluate the physicochemical properties, irritation potential (HET-CAM assay), and broad-spectrum antimicrobial activity of FuCho extracted from these novel Brazilian species. By coupling biodiversity exploration with industrial residue valorization, this study directly aligns with Sustainable Development Goal 12 (Responsible Consumption and Production), offering an innovative, eco-friendly approach for biopolymer applications in health and material sciences.

## 2. Results and Discussion

### 2.1. Biomass and FuCho Production by Mucorales Fungi: Screening Assay

The production of biomass and fungal chitosan (FuCho) during the screening of nineMucorales strains cultivated under standardized fermentation conditions (4.0% (*v*/*v*) CSL and 2.0% (*v*/*v*) GLY) is presented in Table 1.

Significant variability in biomass production (ranging from 4.17 to 10.61 g/L) and FuCho yield (ranging from 17.88 to 126.56 mg/g) was observed among the evaluated Mucorales strains. Notably, *A. caatinguensis* and *C. elegans* exhibited the highest FuCho yields, outperforming values previously reported for Mucorales grown in other waste-based or synthetic media [9,13,14,15,16,17]. Consequently, *A. caatinguensis* and *C. elegans* were selected for optimization via a $2^{2}$ central composite rotatable design (CCRD) to evaluate the combined influence of CSL and GLY concentrations on FuCho production and to compare biopolymer yields between the two species of Mucorales.

The sustainable culture medium composed of CSL and GLY proved to be an effective source of carbon and nitrogen for FuCho production via microbial fermentation (Table 1), particularly for novel Mucorales species (*A. caatinguensis*, *A. aguabelensis*, *B. constricta*, *L. brasiliensis*, and *M. pernambucoensis*) whose biopolymer production potential had not been previously evaluated.

Optimizing fermentation conditions using statistical approaches is crucial for understanding how environmental variables influence FuCho synthesis. Factors such as carbon and nitrogen source ratios, incubation time, agitation speed, aeration, pH, and temperature are routinely explored to maximize FuCho yields for industrial scale-up [18,19,20]. Screening diverse Mucorales fungi under standardized conditions represents a key first step, allowing for the selection of high-yielding strains before subjecting them to multi-variable optimization.

The variation in FuCho production among strains reflects species-specific metabolic adaptations and physiological strategies for synthesizing cell wall polysaccharides, particularly through the activation of chitin deacetylase (CDA), the key enzyme catalyzing the enzymatic deacetylation of fungal chitin [21]. Furthermore, even strains belonging to the same species or genus isolated from distinct environmental niches can exhibit contrasting FuCho accumulation capabilities [16,20,22,23], as evidenced by our findings.

### 2.2. Biomass and FuCho Production by A. caatinguensis URM 7156 and C. elegans UCP 1306: CCRD 2^2^ Assay

The production of biomass and FuCho by *A. caatinguensis* and *C. elegans* was influenced by the CSL and GLY concentrations in the culture medium, as determined in the 2^2^ central composite rotatable design (CCRD) assay (Table 2).

Four empirical equations were derived from the experimental biomass and FuCho yields obtained for *A. caatinguensis* and *C. elegans* through the calculation of regression coefficients, expressing the effects of the independent variables—CSL ($x_{1}$) and GLY ($x_{2}$) concentrations—and their interaction ($x_{1}x_{2}$) on the response variables. Equations (1) and (2) describe the biomass and FuCho yields produced by *A. caatinguensis*, while Equations (3) and (4) describe the biomass and FuCho yields for *C. elegans*:

Y_Biomass (g/L)_ = 10.66 + 0.06x_1_ + 2.20x_2_ + 1.40x_1_x_2_ − 1.47x_1_^2^ − 0.96x_2_^2^ (1)

Y_FuCho (mg/g)_ = 119.86 + 1.30x_1_ + 11.11x_2_ + 5.50x_1_x_2_ − 40.90x_1_^2^ − 31.49x_2_^2^ (2)

Y_Biomass (g/L)_ = 9.70 + 3.36x_1_ + 1.23x_2_ + 0.99x_1_x_2_ − 0.04x_1_^2^ − 0.22x_2_^2^ (3)

Y_FuCho (mg/g)_ = 85.52 − 10.29x_1_ − 16.58x_2_ + 4.54x_1_x_2_ + 11.13x_1_^2^ − 11.84x_2_^2^ (4)

**Figure 1:**
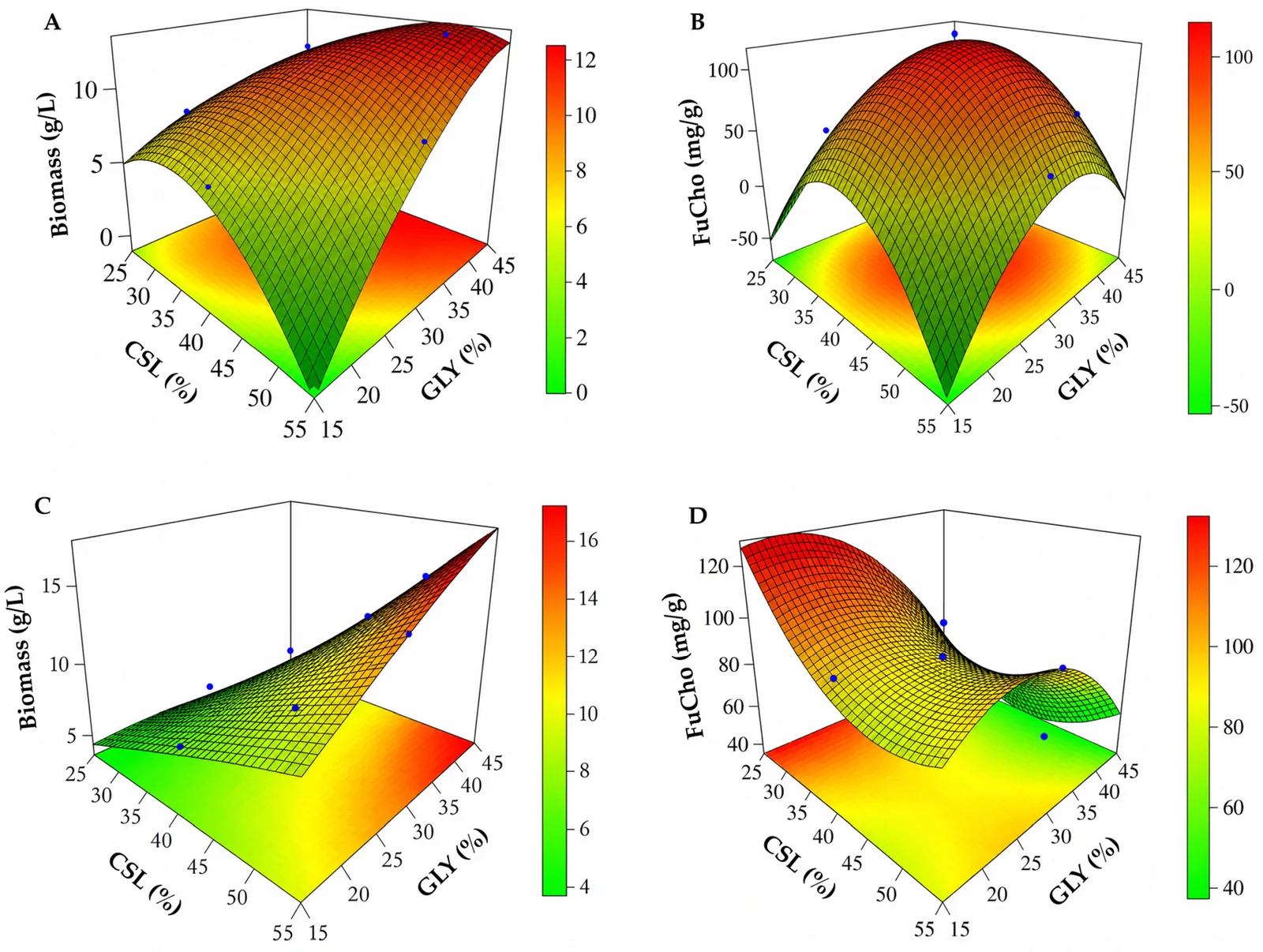
Three-dimensional response surface plots showing the effects of corn steep liquor (CSL; %, *v*/*v*) and glycerol (GLY; %, *v*/*v*) on: biomass production (**A**) and chitosan yield (**B**) by *Absidia caatinguensis* URM 7156; and biomass production (**C**) and chitosan yield (**D**) by *Cunninghamella elegans* UCP 1306.

The results showed higher biomass production by both *A. caatinguensis* and *C. elegans* in run 4, which corresponds to the highest CSL and GLY concentrations (Table 2), as further illustrated in the three-dimensional response surface plots (Figure 1) and predicted by Equations (1) and (3) (Figure 1A,C).

However, the highest FuCho yields were not directly proportional to fungal biomass for either strain, consistent with the observations from the screening assay (Section 2.1). Previous studies have similarly reported that there is no directly linear relationship between mycelial growth and biopolymer synthesis in fungi, suggesting that fermentation conditions distinctly influence biomass accumulation and polysaccharide biosynthesis [10,18,19,22].

The highest FuCho yields for *A. caatinguensis* and *C. elegans* were achieved in run 9 (128.26 mg/g) and run 5 (164.92 mg/g), respectively. For *C. elegans*, the maximum FuCho yield (164.92 mg/g) obtained in run 5 corresponds to the lower axial level of CSL (1.18% v/v). As indicated by the response surface model (Equation (4) and Figure 1D), lower nutrient concentrations appear to favor specific biopolymer accumulation in this strain. While this value highlights the remarkable capacity of *C. elegans* to produce FuCho under minimal nutrient supplementation, it indicates that the true stationary optimum may extend beyond the lower boundary of the present experimental design, warranting further range expansion in future studies.

From an industrial perspective, considering the biomass densities obtained under these conditions (10.07 g/L and 5.21 g/L, respectively), these values translate into volumetric FuCho yields of 1.29 g/L and 0.85 g/L, respectively. These results confirm the scalability of the process, balancing specific chitosan accumulation with biomass accumulation. Although Run 4 achieved a higher volumetric biomass concentration (15.27 g/L), corresponding to an estimated volumetric FuCho production from *C. elegans* of approximately 1.17 g/L, Run 5 exhibited a substantially higher specific polymer accumulation (164.92 mg/g versus 76.40 mg/g in Run 4). The experimental design was intentionally optimized to maximize the specific intracellular accumulation of FuCho (mg/g biomass) rather than overall biomass production. From the perspectives of downstream processing and green engineering, recovering FuCho from biomass with a higher specific polymer content (Run 5) markedly decreases the consumption of alkaline and acidic reagents required per gram of the target polymer. Consequently, prioritizing specific polymer accumulation minimizes chemical inputs during cell wall extraction, thereby enhancing process sustainability and aligning with the principles of green chemistry and the circular economy.

The highest FuCho yield by *A. caatinguensis* was obtained at intermediate levels of CSL and GLY (runs 9–12, center point). Conversely, lower concentrations of CSL and GLY resulted in higher FuCho production by *C. elegans* (Figure 1D, Equation (4)). The coefficients of determination ($R^{2}$) for the models comparing experimental biomass and FuCho yields with CSL and GLY concentrations were 0.96 and 0.97 for *A. caatinguensis*, and 0.97 and 0.95 for *C. elegans*, respectively.

Biomass and FuCho production were positively influenced by the high nitrogen (6.49%) and carbon (37.77%) content of CSL, as well as GLY as an abundant carbon source (65.19%), as determined by elemental analysis. Previous studies have evaluated these by-products separately as economical C and N sources for fungal growth and biopolymer production [7,10,24]. Furthermore, when cultivated in culture media with specific combinations of CSL and GLY, the fungal strains demonstrated increased yields of both biomass and FuCho. Fermentation variables—particularly carbon and nitrogen availability, the C:N ratio, and trace mineral elements—exert a strong influence on microbial growth dynamics. In recent years, several studies have confirmed that FuCho yield depends on the fungal growth phase, cultivation methodology (Table 3), and the intrinsic physiological characteristics of the producing strain [25,26]. Cultivating fungi in alternative media derived from nutrient-rich agro-industrial by-products consistently provides higher biopolymer yields compared to standard synthetic media.

Previous studies (Table 3) illustrate the diversity of FuCho yields obtained across different fungal strains under specific culture conditions. Consequently, multivariate optimization approaches continue to be essential for clarifying how operating variables (fungal species, nutrient composition, pH, temperature, aeration, and agitation speed) interact during microbial chitosan production, ultimately driving competitive alternatives to crustacean-derived chitosan.

### 2.3. Characterization of Fungal Chitosan (FuCho): Infrared Spectroscopy, X-Ray Diffraction and Thermal Analysis

**Figure 2:**
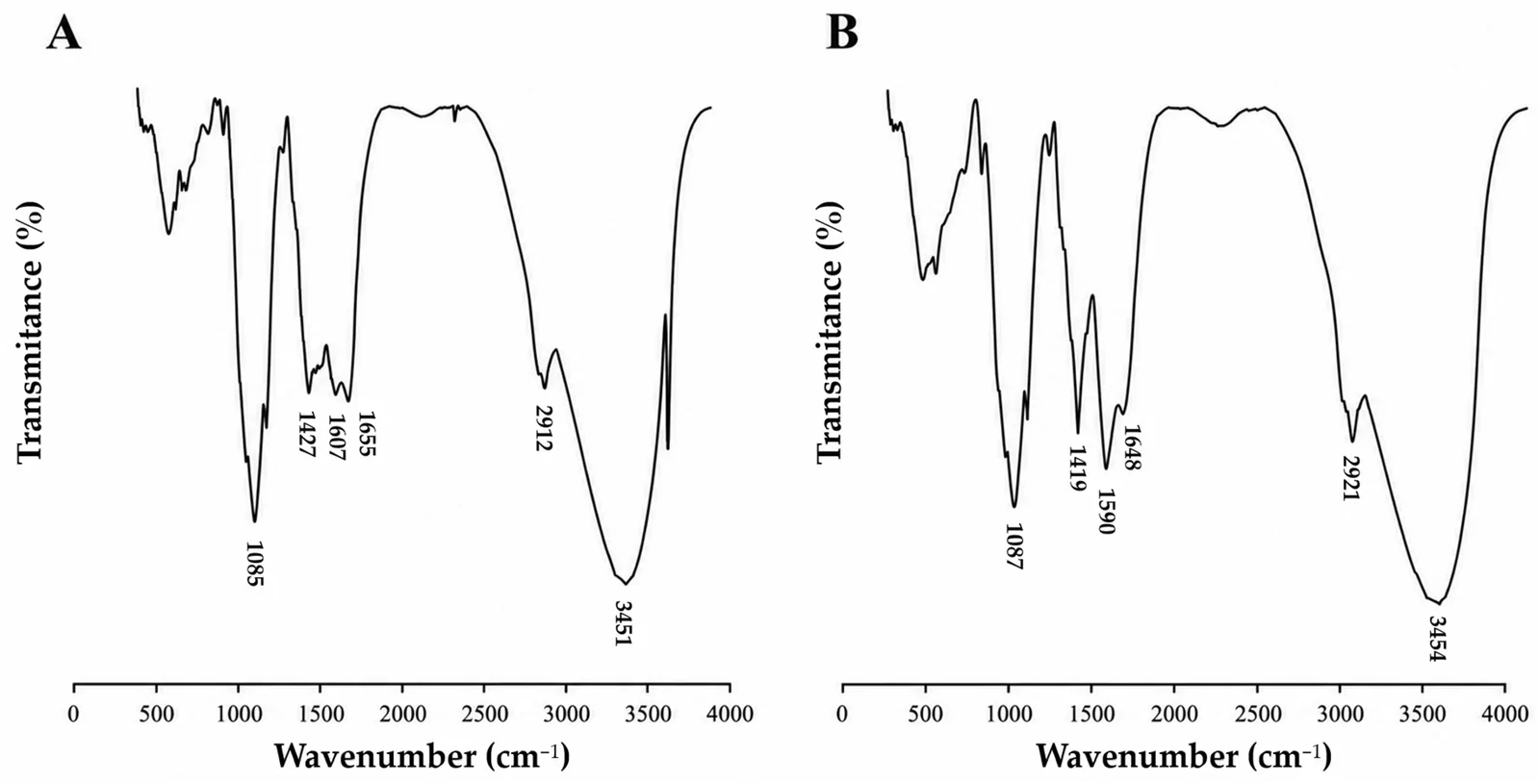
Fourier-transform infrared (FTIR) spectrum of fungal chitosan (FuCho) from *Cunninghamella elegans* UCP 1306 (**A**) and *Abisidia caatinguensis* URM 7156 (**B**).

The FTIR spectra of FuCho extracted from *C. elegans* and *A. caatinguensis* (Figure 2A,B) exhibited characteristic absorption bands consistent with those reported in previous studies for fungal chitosan [10,25,28] and crustacean-derived chitosan [32,33,34].

The FTIR spectra of FuCho from *C. elegans* and *A. caatinguensis* displayed key functional group vibrations, with values reported respectively for each strain. A broad band was observed corresponding to OH and NH stretching vibrations (3451 cm^−1^ for *A. caatinguensis* and 3454 cm^−1^ for *C. elegans*), along with aliphatic C-H stretching (2921 cm^−1^ and 2912 cm^−1^). In addition, characteristic absorption bands were identified for C=O-NHR stretching in amide I (1648 cm^−1^ and 1655 cm^−1^), N–H deformation in the CONH plane for amide II (1590 cm^−1^ and 1607 cm^−1^), C–N bond stretching combined with $-{\mathrm{CH}}_{3}$ wagging in amide III (1419 cm^−1^ and 1427 cm^−1^), and the antisymmetric stretching vibration of the C-O-C glycosidic linkages (1087 cm^−1^ and 1085 cm^−1^).

The deacetylation process converts N-acetylglucosamine units into glucosamine units bearing free primary amino groups ($-{\mathrm{NH}}_{2}$). The band near $1655{\;\mathrm{cm}}^{-1}$ (amide I, C=O stretching) confirms the presence of residual N-acetyl groups within the biopolymer backbone.

The degree of deacetylation (DD) calculated from FTIR spectra was 75% for *C. elegans* FuCho and 85% for *A. caatinguensis* FuCho, both well above the minimum threshold of 60% required to define chitosan and ensure solubility in dilute organic acid solutions [29]. These values align with literature data (Table 3), as DD is known to vary depending on fungal species and extraction parameters [22].

**Figure 3:**
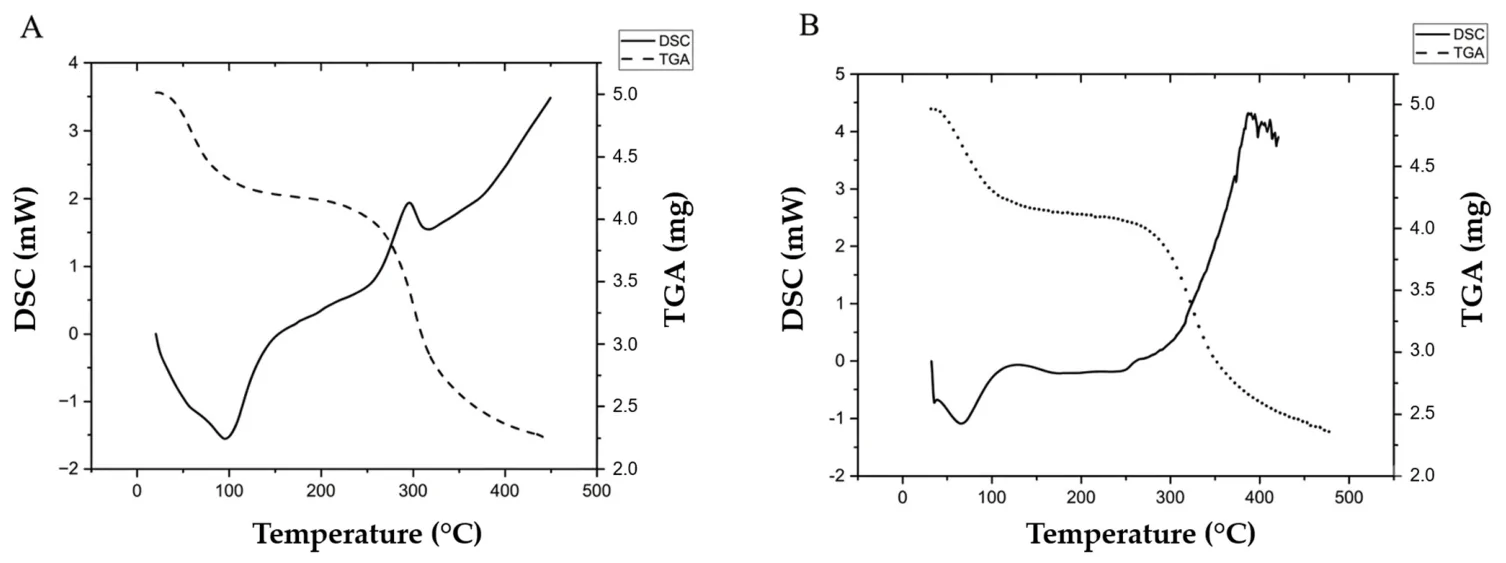
Thermogravimetric analysis (TGA) and differential scanning calorimetry (DSC) thermograms of fungal chitosan (FuCho) from *Cunninghamella elegans* UCP 1306 (**A**) and *Abisidia caatinguensis* (**B**).

Thermal characterization of FuCho from *C. elegans* and *A. caatinguensis* (Figure 3) was conducted using thermogravimetric analyses (TGA) and differential scanning calorimetry (DSC). The DSC thermograms revealed an initial endothermic event beginning around 20 °C and peaking near 97 °C, corresponding to the evaporation of physically adsorbed water. A second exothermic event occurred at higher temperatures (295 °C until 315 °C), associated with the thermal decomposition and carbonization of the biopolymer chain [29].

These DSC thermal events strongly agree with the two-step degradation behavior observed in the TGA curves (Figure 3A,B). The primary amino and hydroxyl groups in chitosan readily adsorb moisture; this bound water is lost below 100 °C for both FuCho during the first weight-loss stage. The second degradation stage, initiating around 180 °C and extending to approximately 438 °C (endset temperature), reflects pyrolytic cleavage of glycosidic bonds and polymer backbone degradation, culminating in a carbonaceous residue, as reported in earlier studies [3,28,35].

**Figure 4:**
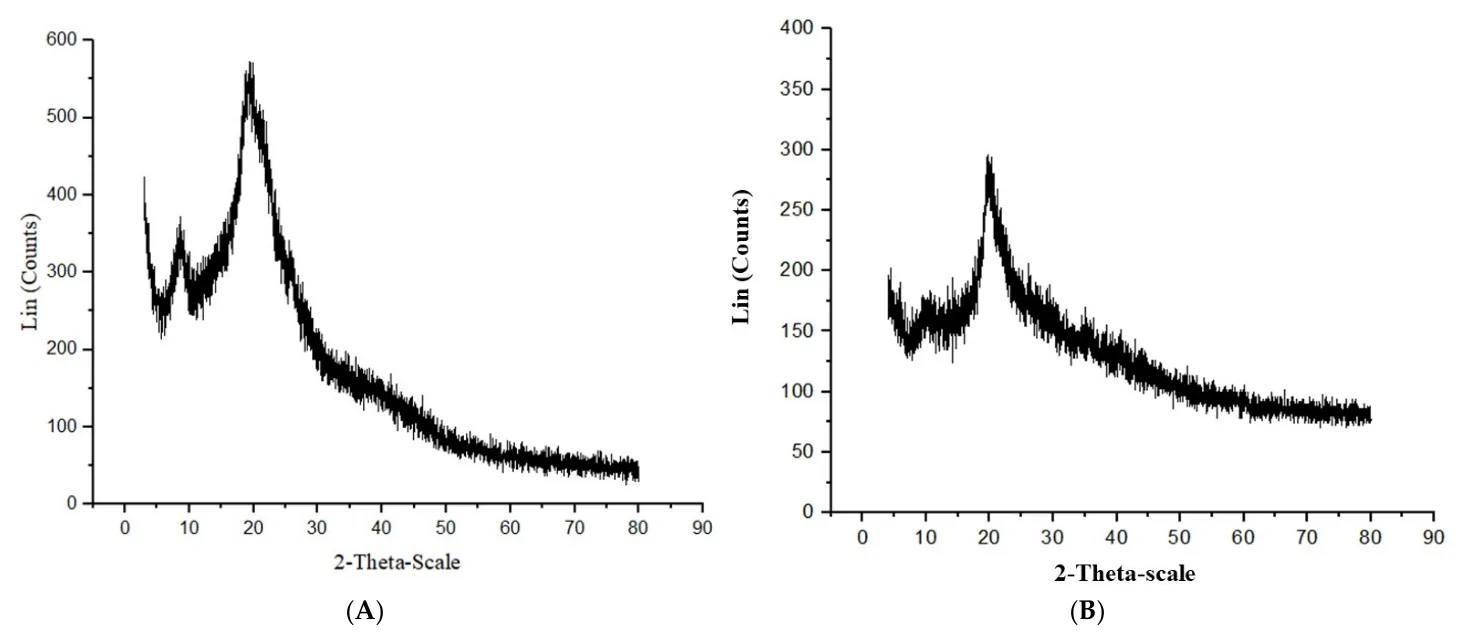
X-ray diffraction (XRD) pattern of fungal chitosan (FuCho) from *Cunninghamella elegans* UCP 1306 (**A**) and *Abisidia caatinguensis* (**B**).

X-ray diffraction (XRD) patterns (Figure 4) exhibited two characteristic crystalline reflection peaks at 2θ ≈ 9.0° and 20.0° with strong Bragg reflections, corroborating earlier findings [3,19,36]. These diffraction peaks correspond to the organized semi-crystalline reticular structure of chitosan. Furthermore, the absence of diffraction peaks at 2θ = 5.0–6.0°—which are characteristic of native chitin—confirms effective deacetylation. Furthermore, the absence of diffraction peaks at 2θ = 5.0–6.0°—which are characteristic of native chitin—confirms effective deacetylation. The relative scattering intensities at 2θ ≈ 9.0–10.0° and 19–20.0° reflect the amorphous and crystalline domains used to calculate the crystallinity index (CI) [3,19,36]. Fungal chitosan generally exhibits lower crystallinity than crustacean chitosan, enhancing the accessibility of free primary amino groups, increasing sorption capacity, and improving solubility in acidic media. The CI values calculated for FuCho from *C. elegans* (36.74%) and *A. caatinguensis* (37.93%) are comparable to values reported for other fungal [13,15,19] and marine chitosans [36].

The viscosity-average molecular weight ($M_{v}$) was calculated as 2.0 × 10^3^ g/mol for *C. elegans* FuCho and 1.6 × 10^3^ g/mol for *A. caatinguensis* FuCho, classifying both as low-molecular-weight biopolymers. The harsh extraction protocol—utilizing autoclaving and high temperatures—likely induced partial depolymerization through cleavage of β-(1-4)-glycosidic linkages. Low-*M_v_* chitosans with high DD offer distinct advantages: increased cationic charge density and enhanced aqueous solubility at physiological pH, which promote cellular membrane interaction and broad-spectrum antimicrobial efficacy [3,4,15].

### 2.4. Morphological Characterization of Fungal Chitosan (FuCho) Extracted from the Biomass of Absidia caatinguensis and from Cunninghamella elegans by Scanning Electron Microscopy (SEM)

**Figure 5:**
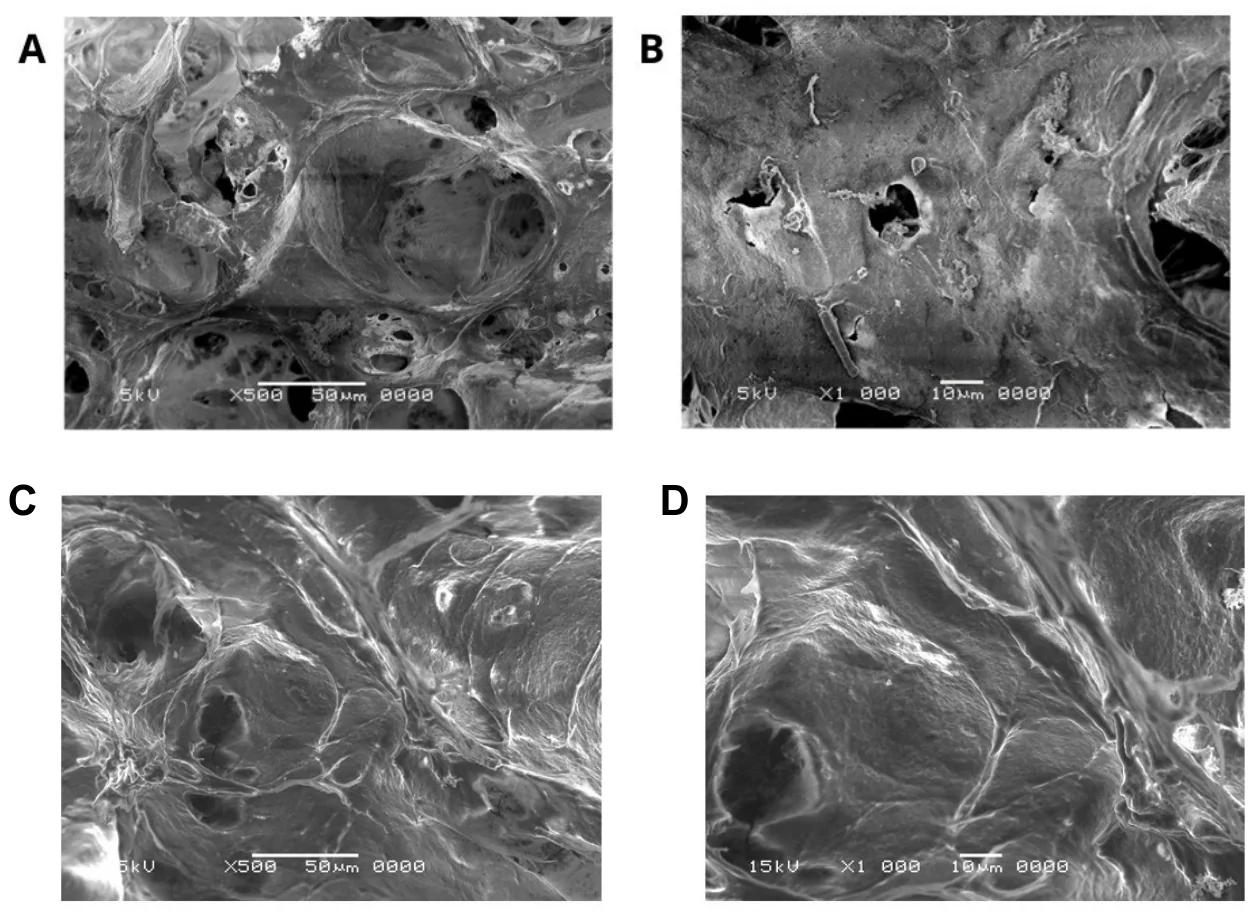
Scanning electron microscopy (SEM) micrographs of fungal chitosan (FuCho) extracted from *Cunninghamella elegans* UCP 1306 at 500× (**A**) and 1000× magnification (**B**); and from *Absidia caatinguensis* URM 7156 at 500× (**C**) and 1000× magnification (**D**). Scale bars = 50 µM (500×) and 10 µM (1000×).

Scanning electron microscopy (SEM) was used to evaluate the surface morphology of FuCho extracted from *C. elegans* and *A. caatinguensis* (Figure 5). The morphological structure of both FuCho samples lacked microfibrillar arrangements, exhibiting instead an amorphous, dense, compact, and rough surface with flat, irregular, and piecemeal regions, with no visible recognizable spatial patterns. Similar flat and piecemeal surface characteristics have been previously reported for fungal chitosans [29,34]. In contrast, commercial crustacean-derived chitosan typically displays a more homogeneous, well-organized morphology with a porous or lamellar/fibrillar matrix resulting from the aggressive chemical demineralization and deproteinization processes required to strip the marine mineralized exoskeleton [36].

The absence of rigid, fibrillar structures in FuCho aligns with its lower crystallinity index (36.74% and 37.93% for *C. elegans* and *A. caatinguensis*, respectively), a characteristic structural feature of fungal biopolymers that enhances the accessibility of free primary amino groups ($-{\mathrm{NH}}_{2}$). This structural flexibility and predominantly amorphous nature not only contribute to higher solubility in dilute acidic media, but also facilitate stronger ionic interactions with microbial cell membranes, thereby supporting the enhanced biological and antimicrobial performance observed for FuCho [3,19].

### 2.5. Irritation Potential Assessment of Fungal Chitosan (FuCho) Extracted from the Biomass of Absidia caatinguensis and from Cunninghamella elegans Using the Hen’s Egg Test–Chorioallantoic Membrane (HET-CAM)

Assessing the toxicity of chemical substances and finished products remains a challenge for regulatory agencies and the scientific community, driving the demand for predictive methodologies that protect of human health and the environment while minimizing the ethical and economic impacts of in vivo testing. In this context, the adoption of New Approach Methodologies (NAMs) has enhanced the predictive capacity of toxicological assessments and contributed to replacing animal testing. Among these alternative approaches, the Hen’s Egg Test–Chorioallantoic Membrane (HET-CAM) assay stands out as a low-cost reliable in ovo model widely used to evaluate acute vascular irritation potential [37,38].

The HET-CAM assay evaluates local compatibility with mucosal membranes because the chick embryo chorioallantoic membrane contains a fully formed vascular network of veins and capillaries, providing a suitable alternative to the in vivo Draize rabbit eye test [37,38,39,40,41]. As an in ovo alternative method conducted prior to the final third of embryonic development (typically on incubation days 9–10), the HET-CAM test falls outside the regulatory scope of animal experimentation under Directive 2010/63/EU [38]. It is recognized by EURL ECVAM as a relevant non-animal approach for ocular irritation testing and can be integrated into Integrated Approaches to Testing and Assessment (IATA) [41], although it has not yet been formally adopted as an OECD Test Guideline for standalone regulatory classification [37]. Furthermore, HET-CAM is a rapid, sensitive, cost-effective, and robust qualitative and quantitative assay that serves as an intermediate model bridging in vitro and in vivo test systems [37,39].

**Figure 6:**
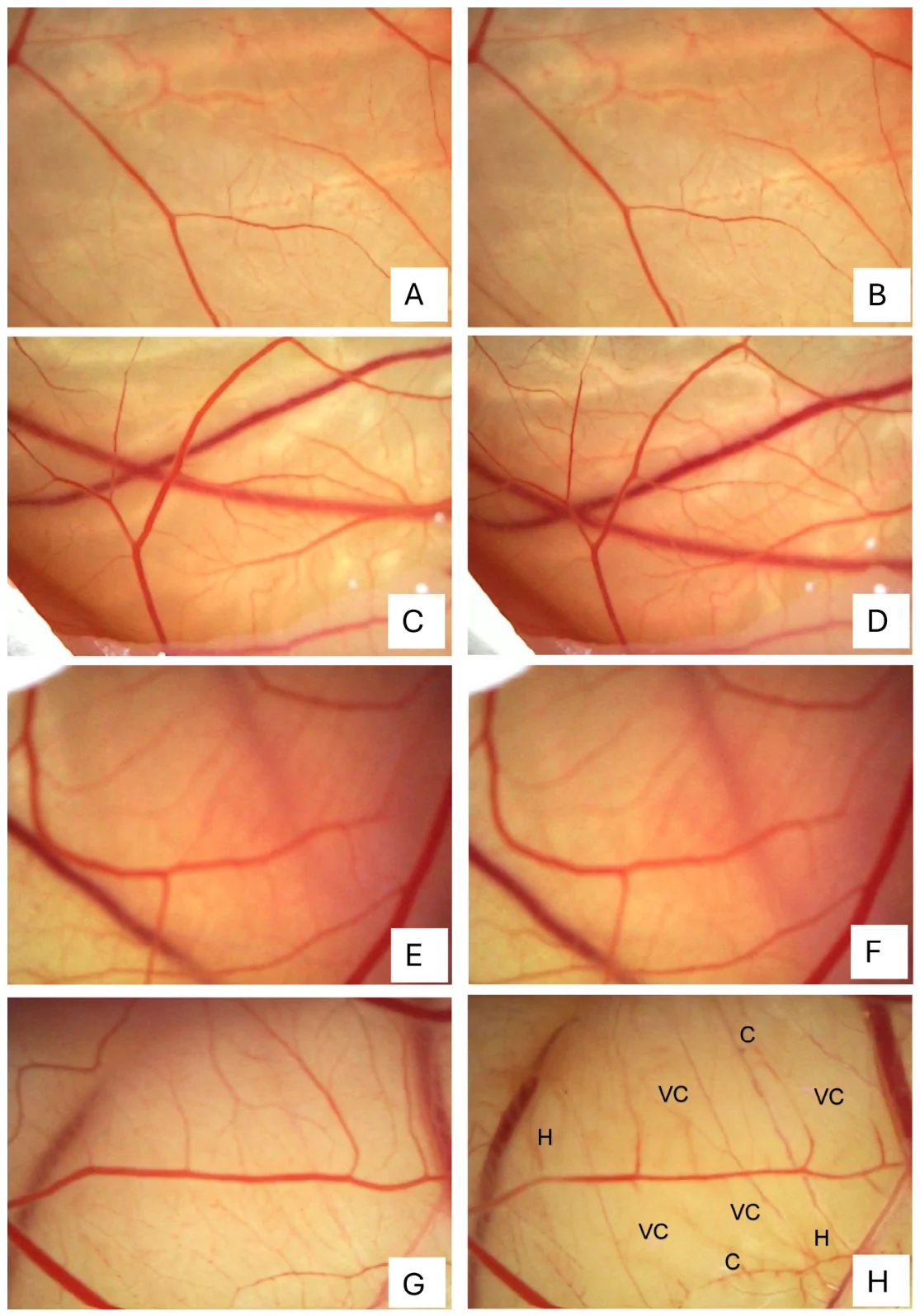
Evaluation of acute irritation potential using the Hen’s Egg Test–Chorioallantoic Membrane (HET-CAM) assay on 8-day-old fertilized hen eggs, observed up to 5 min (300 s) for vascular effects (vasoconstriction-VC, hemorrhage-H, and coagulation-C). Images show the membrane at time zero (prior to application) and after 300 s of exposure for: (**A**,**B**) negative control (0.9% w/v NaCl solution); (**C**,**D**) fungal chitosan (FuCho) from *Cunninghamella elegans*; (**E**,**F**) fungal chitosan (FuCho) from *Absidia caatinguensis*; and (**G**,**H**) positive control (1% w/v sodium lauryl sulfate, SLS).

The HET-CAM results demonstrated that the negative control (0.9% w/v NaCl, Figure 6A,B), as well as FuCho extracted from *C. elegans* (Figure 6C,D) and *A. caatinguensis* (Figure 6E,F), were non-irritating. All three treatments yielded an Irritation Score (IS) of 0.0, displaying no signs of inflammatory or vascular damage (such as vasoconstriction, hemorrhage, or coagulation) throughout the 300 s observation period. In contrast, the positive control (1% *w*/*V* sodium lauryl sulfate, SLS) produced severe irritation with an IS of 17.74 ± 0.4. This severe irritant reaction was characterized by a rapid onset of vascular damage, including vasoconstriction (6.0 ± 1.0 s), coagulation (63.0 ± 3.0 s), and hemorrhage (48.0 ± 3.0 s) (Figure 6G,H). Among the recorded irritation parameters, vasoconstriction was the most frequent event, followed by coagulation and hemorrhage.

These HET-CAM findings strongly indicate that FuCho possesses a safe vascular compatibility profile with negligible irritation potential, in agreement with previously reported data [39,42]. Additional cytotoxicity, genotoxicity, and mutagenicity assays are recommended to fully establish their overall safety profile for biomedical and topical applications.

### 2.6. Determination of the Minimum Inhibitory Concentration (MIC) of FuCho Extracted from the Biomass of Absidia caatinguensis and from Cunninghamella elegans

Chitosan (FuCho) extracted from the biomass of *A. caatinguensis* and *C. elegans* grown in CLS-GLY culture medium inhibited the growth of all tested pathogenic species of bacteria and *Candida*, with minimum inhibitory concentration (MIC) values presented in Table 4. Among the strains tested, *Enterococcus faecium* demonstrated the highest level of resistance to both chitosans, exhibiting MIC values of 5 mg/mL for *C. elegans* FuCho and 4 mg/mL for *A. caatinguensis* FuCho. This was followed by *Candida krusei* and *Candida albicans*, which both displayed MICs of 4.0 mg/mL (*C. elegans*) and 3.0 mg/mL (*A. caatinguensis*). Both FuCho samples exhibited identical MIC values for *Staphylococcus epidermidis* (2 mg/mL), *Streptococcus oralis* (1 mg/mL), *Streptococcus mutans* (1 mg/mL), *Pseudomonas aeruginosa* (2 mg/mL), and *Escherichia coli* (2 mg/mL). For *Staphylococcus aureus*, *Streptococcus salivarius*, and *Streptococcus gordonii*, FuCho from *A. caatinguensis* exhibited lower MIC values than that from *C. elegans* (Table 4).

The differences in MIC values observed between *A. caatinguensis* and *C. elegans* chitosans can be attributed, in part, to their distinct degrees of deacetylation (DD), as both polymers possess similar low molar masses (1.6 × 10^3^ g/mol and 2.0 × 10^3^ g/mol, respectively). Chitosan extracted from *A. caatinguensis* has a higher DD of 85%, whereas that from *C. elegans* has a DD of 75%. Czajkowska et al. [43] demonstrated that the antimicrobial potency of soluble chitosans (DD > 60%) is predominantly driven by the degree of deacetylation rather than molecular weight. Specifically, a linear inverse relationship was observed, with each 1% increase in DD yielding an average reduction of 0.040 in log(MIC), whereas molecular weight showed no statistically significant influence on biocidal performance.

The enhanced antimicrobial activity observed for the FuCho samples (75% and 85% DD) is likely associated with a higher density of free amino groups (NH_2_) available for protonation under the experimental conditions (pH 5.5–6.0). At a pH below the pKa of chitosan (approx. 6.3–6.5), a substantial proportion of these amino groups remains protonated (NH_3_^+^), increasing the cationic character of the polymer. This higher positive charge density promotes stronger electrostatic interactions with negatively charged cell surface components (such as teichoic and lipoteichoic acids in Gram-positive bacteria, or lipopolysaccharides in Gram-negative bacteria), leading to membrane disruption, increased permeability, intracellular leakage, and cell death. Nevertheless, other physicochemical properties, including molecular weight distribution and polymer conformation in solution, may also contribute to the observed variations [26,43,44,45,46].

Chitosan is widely recognized as a broad-spectrum antimicrobial agent effective against bacteria, molds, and yeasts [43]; however, fungi generally exhibit higher resistance to the polymer and its derivatives. Regarding bacteria, debates persist concerning its comparative efficacy against Gram-negative versus Gram-positive strains [44]. Although the exact mechanism of action remains to be fully elucidated, three primary pathways have been proposed: (1) surface membrane destabilization via electrostatic interactions between protonated amino groups (NH_3_^+^) and anionic cell wall components; (2) intracellular accumulation of low-to-medium molar mass chitosan, which alters DNA/RNA transcription and inhibits protein synthesis [47]; and (3) chelation of essential trace elements and microelements, thereby limiting nutrient availability and suppressing microbial growth [44].

### 2.7. Effect of FuCho Extracted from the Biomass of Absidia caatinguensis and from Cunninghamella elegans on Bacterial Morphology and Membrane Integrity by Confocal Microscopy

**Figure 7:**
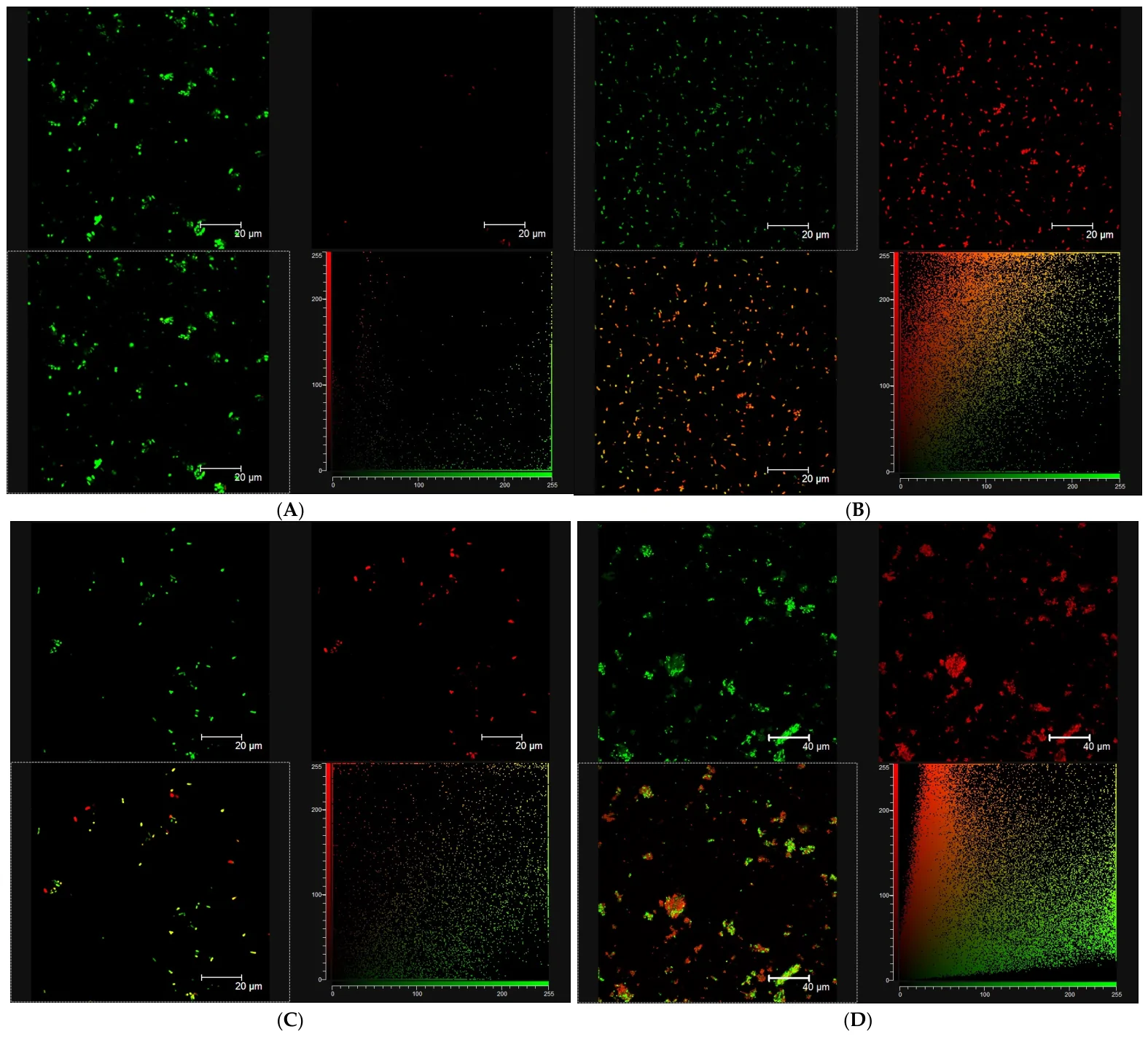
Assessment of *Staphylococcus aureus* membrane integrity and morphology by Confocal Laser Scanning Microscopy (CLSM) using SYTO 9 (green, intact membranes) and propidium iodide (PI, red, damaged membranes) dual staining, accompanied by 2D fluorophore colocalization scatter plots (bottom-right panel of each group): (**A**) untreated control cells showing intact viability; (**B**) positive control (70% *v*/*v* isopropyl alcohol) exhibiting severe membrane compromise; (**C**) cells treated with *Cunninghamella elegans* FuCho at MIC showing cell injured and detachment; and (**D**) cells treated with *Absidia caatinguensis* FuCho at MIC displaying extensive cell aggregation and entrapment of live and dead bacteria. Scale bars: 20 µm (**A**–**C**) and 40 µm (**D**).

To evaluate the impact of FuCho on cell viability and membrane integrity, *Staphylococcus aureus* cells exposed to minimum inhibitory concentrations (MIC) for 30 min were analyzed using dual-fluorescent staining with SYTO 9 and propidium iodide (PI) via Confocal Laser Scanning Microscopy–CLSM (Leica Microsystems, Mannheim, Germany) (Figure 7).

Untreated control *S. aureus* cells displayed a high density of well-distributed, intact bacteria characterized by strong green fluorescence (SYTO 9) and negligible red fluorescence (PI) (Figure 7A). The corresponding 2D fluorophore colocalization plot confirmed intact plasma membranes with minimal baseline PI uptake under physiological conditions. In contrast, *S. aureus* cells exposure to 70% *v*/*v* isopropyl alcohol (positive control) induced membrane-compromised, marked by a widespread shift to intense red fluorescence and a massive pixel shift toward the *Y*-axis in the colocalization plot (Figure 7B).

Treatment with *C. elegans* FuCho at MIC caused a drastic reduction in visible *S. aureus* cell density within the field of view (Figure 7C). The few remaining scattered cells exhibited low fluorescence intensity, resulting in a depleted 2D scatter plot. This pattern suggests that *C. elegans* FuCho promotes rapid cell lysis and detachment of damaged bacteria from the substrate. Conversely, *A. caatinguensis* FuCho demonstrated a distinct mechanism dominated by extensive cellular aggregation (Figure 7D). Large, dense bacterial clusters were formed, exhibiting strong green labeling at the periphery and prominent interior red fluorescence. The corresponding colocalization plot displayed a clear fan-like distribution, indicating the co-entrapment of both viable and membrane-compromised cells within a compact polymeric matrix. These findings highlight the potent flocculating and bactericidal properties of *A. caatinguensis* FuCho against Gram-positive pathogens.

**Figure 8:**
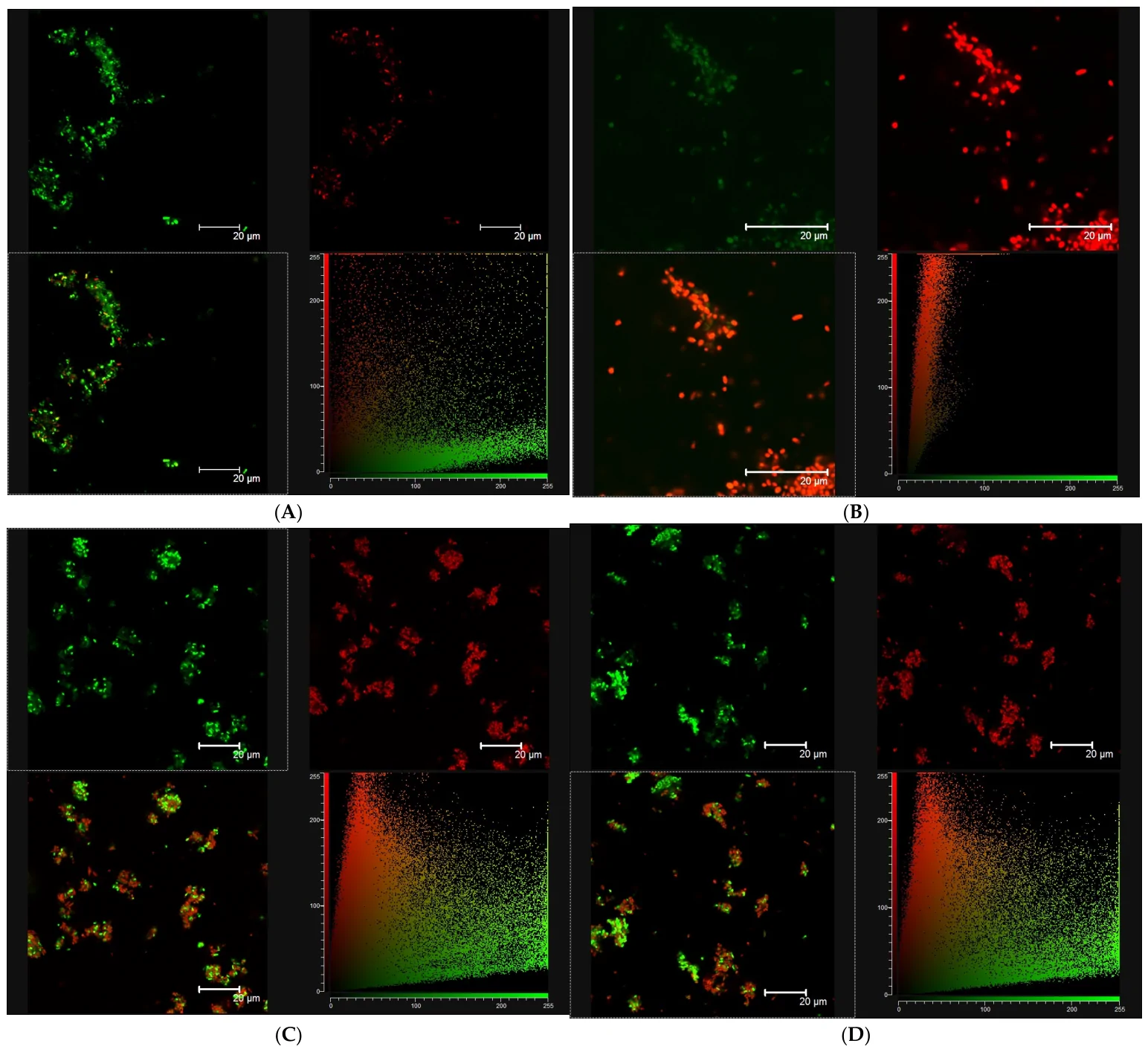
Assessment of *Escherichia coli* membrane integrity and morphology by Confocal Laser Scanning Microscopy (CLSM) using SYTO 9 (green, intact membranes) and propidium iodide (PI, red, damaged membranes) dual staining, accompanied by 2D fluorophore colocalization scatter plots (bottom-right panel of each group): (**A**) untreated control cells displaying natural viability clusters and baseline green emission; (**B**) positive control (70% *v*/*v* isopropyl alcohol) showing severe membrane solubilization and vertical shift to *Y*-axis; (**C**) cells treated with *Cunninghamella elegans* FuCho at MIC demonstrating extensive aggregation, yellow-orange composite emission, and diagonal colocalization; and (**D**) cells treated with *Absidia caatinguensis* FuCho at MIC showing severe cell depletion, membrane-compromised, and detachment. Scale bars: 20 µm.

The effect of fungal chitosan on Gram-negative bacterial morphology and membrane integrity was further evaluated using *Escherichia coli* exposed to FuCho at MIC for 30 min (Figure 8).

Untreated control *E. coli* cells exhibited natural bacterial clusters characterized by well-defined green fluorescence (SYTO 9) and a negligible baseline red signal (PI), confirming intact outer and cytoplasmic membranes (Figure 8A). The corresponding colocalization scatter plot showed a dense population concentrated along the base of the *X*-axis (green channel), quantifying the dominance of intact cells and the absence of membrane compromise. In contrast, treatment with 70% *v*/*v* isopropyl alcohol (positive control) induced near-complete loss of green fluorescence, leaving only faint residual cellular outlines, accompanied by massive red labeling across the remaining bacterial population (Figure 8B). In the colocalization plot, pixels underwent a radical vertical shift toward the *Y*-axis (red channel), reflecting extensive lipid membrane compromised, loss of SYTO 9 via cell injured, and unhindered influx of PI.

When exposed to *C. elegans* FuCho at MIC, *E. coli* underwent extensive cellular aggregation, forming large, dense bacterial clusters (Figure 8C). The merged image displayed strong yellow-orange composite fluorescence, while the colocalization plot exhibited a wide fan-like pattern spanning the diagonal. This pattern indicates simultaneous uptake of both dyes and substantial membrane destabilization resulting from polymer-mediated bacterial entrapment. Conversely, treatment with *A. caatinguensis* FuCho at MIC led to a drastic depletion of bacterial density within the field of view, with no aggregate formation (Figure 8D). Only a few isolated, viable cells remained attached to the substrate, generating a sparse cloud of points in the lower region (*X*-axis) of the colocalization plot. This marked reduction in cell density suggests that *A. caatinguensis* FuCho triggers rapid cell injured accompanied by cell detachment or severe inhibition of bacterial adhesion, highlighting distinct species-dependent mode-of-action profiles against Gram-negative pathogens.

Overall, confocal laser scanning microscopy confirmed that exposure to FuCho from both *C. elegans* and *A. caatinguensis* at MIC values induced severe membrane compromise in both *S. aureus* (Figure 7C,D) and *E. coli* (Figure 8C,D). This was evidenced by a marked increase in fluorophore emission from green (SYTO 9) to red (propidium iodide), reflecting loss of plasma membrane integrity and widespread cell death. In the merged images, bacterial cells frequently appeared as compact aggregates displaying both green and red fluorescence, suggesting heterogeneous membrane damage within the population. Interestingly, while both polymers exhibited high bactericidal activity, their physical interaction with the cell surfaces varied markedly in a species-dependent manner, triggering either extensive cell aggregation/entrapment or rapid cellular injury accompanied by detachment. A similar shift from viable green-stained populations to dead red-stained cells was previously observed for *Staphylococcus aureus* V329 and *Staphylococcus xylosus* 1007 upon treatment with increasing concentrations of native chitosan and chitosan nanoparticles [48].

The cell viability and membrane integrity test using the fluorescent dyes SYTO^®^9 (green) and PI (red) is based on metabolic characteristics or membrane integrity. Bacteria with damaged membranes are considered nonviable or have exhausted viability based on red staining with propidium iodide. Bacteria with intact membranes are considered viable based on green staining with SYTO9, resulting in differential staining. Cells stained yellow or orange indicate injury to the cell membrane or metabolic alteration indicating that the cells have reduced viability [38].

## 3. Materials and Methods

### 3.1. Fungal Strains and Substrate Characterization

A total of nine *Mucorales* fungal strains, including species newly isolated from soil across different biomes in Pernambuco State, Brazil, were evaluated for biomass and fungal chitosan (FuCho) production:

- Caatinga Biome: *Rhizopus arrhizus* UCP 1295, *Cunninghamella elegans* UCP 1306, *Syncephalastrum racemosum* UCP 1302, and *Cunninghamella phaeospora* UCP 1303 (isolated from São José do Belmonte: $07^{\circ }59^{\prime }52^{\prime \prime }S$, $038^{\circ }37^{\prime }18^{\prime \prime }W$); *Absidia caatinguensis* URM 7156 (Catimbau National Park, Buíque: $08^{\circ }31^{\prime }55.8^{\prime \prime }S$, $037^{\circ }15^{\prime }34.2^{\prime \prime }W$); *Absidia aguabelensis* URM 8213 (Serra do Comunaty, Águas Belas: $09^{\circ }06^{\prime }03.7^{\prime \prime }S$, $037^{\circ }03^{\prime }08.9^{\prime \prime }W$); and *Lichtheimia brasiliensis* URM 6910 (Araripina: $07^{\circ }27^{\prime }58^{\prime \prime }S$, $040^{\circ }24^{\prime }53^{\prime \prime }W$).
- Atlantic Rainforest Biome: *Backusella constricta* URM 7323 (Dois Irmãos Ecological Reserve, Recife: $08^{\circ }00^{\prime }43.2^{\prime \prime }\;S$, $034^{\circ }56^{\prime }40.9^{\prime \prime }\;W$) and *Mucor pernambucoensis* URM 7640 (Serra do Bonito, Bonito: $08^{\circ }28^{\prime }12^{\prime \prime }\;S$, $035^{\circ }43^{\prime }44^{\prime \prime }\;W$).

Strains designated with the UCP prefix were obtained from the Culture Collection of the Nucleus of Research in Environmental Science and Biotechnology (UNICAP, Recife, Pernambuco, Brazil). Strains designated with the URM prefix were provided by the Micoteca URM Culture Collection (Department of Mycology, Federal University of Pernambuco, Recife, Pernambuco, Brazil). Both culture collections are registered with the World Federation for Culture Collections (WFCC). Access to the genetic heritage was registered in the National System for the Management of Genetic Heritage and Associated Traditional Knowledge (SisGen) under code AC60F94.

Corn steep liquor (CSL) and crude glycerol (GLY) were supplied by local food and biorefinery industries (Cabo de Santo Agostinho, Pernambuco, Brazil) and stored at ${4\;}^{\circ }C$ prior to use. Elemental analysis (C, H, and N content, %) of these agro-industrial by-products was performed using an EA 1110 elemental analyzer (Carlo Erba Instruments, Milan, Italy) [25]. Both substrates were used without prior pretreatment as low-cost carbon and nitrogen sources.

### 3.2. Screening Assay and FuCho Extraction

Fermentation experiments were performed in Erlenmeyer flasks containing 150 mL of culture medium (4.0% v/v CSL and 2.0% v/v GLY, adjusted to pH 5.7). Each flask was inoculated with a 1 cm diameter agar plug taken from the edge of a 18 h fungal culture grown on Potato Dextrose Agar (PDA) at ${28\;}^{\circ }C$. Cultures were incubated at ${28\;}^{\circ }C$ and 150 rpm for 96 h [9,13].

Following incubation, biomass was harvested and subjected to FuCho extraction according to the method described by Hu et al. [49] with modifications. Briefly, freeze-dried biomass was deproteinized with 4% (w/v) NaOH solution (1:40 w/v) at $120^{\circ }C$ for 15 min. The alkali-insoluble fraction was recovered by centrifugation (4000×g, 15 min, ${4\;}^{\circ }C$), treated with 2% (v/v) acetic acid (1:100 w/v) at $100^{\circ }C$ for 15 min, and centrifuged again (4000× *g*, 15 min, ${4\;}^{\circ }C$). The resulting supernatant was adjusted to pH 10.0 using 1 M NaOH to precipitate FuCho. The precipitate was collected by centrifugation (4000×g, 15 min, ${4\;}^{\circ }C$), washed with distilled water to neutrality (pH 7.0), freeze-dried, and kept for characterization. All screening assays were conducted in quadruplicate (n=4), and data are expressed as mean ± standard deviation.

### 3.3. Effects of CSL-GLY on the Production of Biomass and FuCho: CCRD 2^2^ Design

Different concentrations of CSL (1.18–6.82%; *v*/*v*) and GLY (0.59–3.41%; *v*/*v*) as alternative sources of nutrients on the production of biomass and FuCho by the two fungi strains that presented the highest FuCho production in screening assay (Section 3.2) were evaluated through a CCRD 2^2^ design. Each assay was carried out in an Erlenmeyer flask with CSL-GLY medium (pH 5.7), inoculated with plugs (1 cm diameter) taken from a fungal culture (grown on PDA at 28 °C for 18 h), and incubated at 28 °C for 96 h under stirring (150 rpm) [15]. After this time, the fungal biomass was washed with distilled water, lyophilized, and used for the extraction of FuCho, as described in Section 3.2.

### 3.4. Characterization of FuCho

Infrared (IR) spectra were obtained using a Bruker 66 IR Spectrometer (Bruker Optics, Ettlingen, Germany) by dispersing 2.0 mg of FuCho in 100 mg of KBr to produce disks of 0.5 mm thickness. The equation: DD (%) = 100 − [(A_1655_/A_3450_) × 115] was used to calculate the degree of deacetylation (DD%) [50], where the absorbance ratio between 1655 and 3450 cm^−1^ was estimated using infrared vibrational spectroscopy (IRVS).

The crystallinity index percentage (CI%) was determined using an X-ray diffractometer, SIEMENS D5000 (Siemens Corporation, Aubrey, TX, USA), with Cu-Kα radiation at λ=1.542 AA, and a scan range of $4^{\circ }\text{–}80^{\circ }$ with a rate of $0.02^{\circ }/\min$. The crystallinity index (CI) was calculated using the following equation: $\mathrm{CI}\;\left(\%\right)=\left[\left(I_{C}-I_{A}\right)/I_{C}\right]\times 100$, where $I_{C}$ and $I_{A}$ correspond to the signal intensities of the crystalline region ($2\theta =20^{\circ }$) and the amorphous region ($2\theta =12^{\circ }$), respectively [9].

Shimadzu model TGA-50WS and Shimadzu model DSC-50WS thermal analysis instruments (Kyoto, Japan) were used for thermogravimetric analysis (TGA) and differential scanning calorimetry (DSC) of FuCho, respectively [51]. The experiment consisted of heating 10 mg of FuCho in an aluminium cup from 0 to 450 °C under a continuous flow of dry nitrogen gas (50 mL/min) at a heating rate of 10 °C/min.

The viscosity-average molecular weight of FuCho (g\mol) was evaluated at 25 °C using a Schott-Geräte viscometer (model AVS-350) equipped with a Cannon-Fenske capillary (*d*_inside_ = 1.01 mm). Flow times (in seconds) were recorded to determine the intrinsic viscosity ([η]). Subsequently, the molecular weight was calculated via the Mark-Houwink equation using the Mark-Houwink-Sakurada parameters for the acetic acid/sodium acetate (HAc/NaAc) solvent system (*K* = 0.076 mL/g and *a* = 0.76) [9].

### 3.5. Scanning Electron Microscopy (SEM) of FuCho

The dried FuCho samples on the aluminium support were subjected to gold/palladium metallization, and their surfaces were observed using a scanning electron microscope Series XL 30 ESEM (JEOL, Tokyo, Japan) equipped with tungsten filaments, at an accelerating voltage of 20 kV [29].

### 3.6. Irritation Potential Test Using Chorioallantoic Membrane (HET-CAM Test)

The Hen’s Egg Test on the Chorioallantoic Membrane (HET-CAM) was performed to evaluate the ocular/vascular irritation potential of FuCho, following the methodology described by Luepke [52] and Directive 2010/63/EU [41]. Fertile White Leghorn chicken eggs (7 days old) were obtained from Mauricéa Alimentos (Aliança, Pernambuco, Brazil). The eggs were incubated for 24 h, at 37 °C and 55% relative humidity. Eggs lacking a clearly visible embryonic vascular network were discarded. The assay was conducted at ambient temperature (23 °C).

The eggshell directly above the air cell was carefully opened, and the inner membrane was hydrated with sterile 0.9% (*w*/*v*) NaCl solution using a sterile cotton swab before being removed, taking care to avoid damaging the underlying microvessels. A volume of 300 µL of each test substance was applied directly onto the chorioallantoic membrane (CAM): 0.9% (*w*/*v*) NaCl solution served as the negative control, 1% (*w*/*v*) sodium lauryl sulfate (SLS) as the positive control, and FuCho (10 mg/mL solubilized in 1% acetic acid, with pH adjusted to 5.5) as the test group. Each group was evaluated using four replicate eggs (*n* = 4). Imaging of the chorioallantoic membrane was performed using a 1000x HD USB digital microscope, model KP-8012 (Knup, Bom Retiro-Santa Catarina, Brazil).

The CAM was monitored for 300 s (5 min) to record the onset time (in seconds) of vascular irritation endpoints: vasoconstriction, hemorrhage, and coagulation. The irritation score (IS, ranging from 0 to 21) was calculated using the equation established by Luepke [52] and Directive 2010/63/EU [41], representing the mean sum of individual endpoint scores across the four replicates. The irritation potential was classified according to the standard scale: 0–0.9 (non-irritant), 1.0–4.9 (slight irritant), 5.0–8.9 (moderate irritant), and 9.0–21.0 (severe irritant).

### 3.7. Determination of the Minimum Inhibitory Concentration (MIC)

The minimum inhibitory concentration (MIC) of fungal chitosans (FuCho) was determined using the broth microdilution method in 96-well flat-bottom microplates. The panel of tested microorganisms included *Staphylococcus aureus* (ATCC 6538), *Staphylococcus epidermidis* (ATCC 35984), *Streptococcus gordonii* (ATCC 35105), *Streptococcus salivarius* (ATCC 7073), *Streptococcus mutans* (NCTC 10449, Oxoid, Basingstoke, UK), *Streptococcus oralis* (ATCC 35037), *Escherichia coli* (ATCC 25922), *Pseudomonas aeruginosa* (ATCC 9028), *Candida albicans* (ATCC 10231), and *Candida krusei* (URM 6391).

Bacterial strains were cultured in Brain Heart Infusion (BHI) broth at 37 °C for 18 h, whereas *Candida* species were cultured in Sabouraud Dextrose Broth (SDB) at 37 °C for 24 h. The microbial strains were then transferred to Petri dishes containing BHI agar (bacteria) and Sabouraud agar (*Candida*) using the streak plate technique to obtain isolated colonies. The microorganisms were incubated at 37 °C for 18 h. After the incubation period, isolated colonies were collected using a sterile disposable loop (standardized volume of 10 µL) and resuspended in a test tube containing 3 mL of 0.85% physiological saline. The microbial pre-inoculum was adjusted to the 0.5 McFarland standard, corresponding to an optical density at 625 nm and 530 nm, respectively, of 0.130 for bacteria (≈$1.5\times 10^{8}$ CFU/mL) and 0.140 for yeasts (≈$1.5\times 10^{6}$ CFU/mL).

After adjustment, the pre-inoculum was diluted 1:100 in BHI broth (bacteria) and Sabouraud broth (*Candida*). A 20 µL aliquot of the standardized diluted inoculum was added to 96-well plates containing 80 µL of culture medium with different concentrations of FuCho (final concentration per well ranging from 0.5 to 6.0 mg/mL). Chlorhexidine solution (final concentration per well from 0.12 to 0.60 mg/mL) served as the positive control. Each well was prepared with a total working volume of 100 µL, consisting of 80 µL of culture medium containing two-fold serial dilutions of FuCho and 20 µL of the standardized microbial inoculum. Thus, the final concentration in the wells was approximately $3\times 10^{5}$ CFU/mL for bacterial strains and $1\times 10^{4}$ CFU/mL for *Candida* strains.

Microplates were incubated at 37 °C for 48 h. Following incubation, resazurin sodium salt solution (Sigma–Aldrich, St. Louis, MO, USA) was added to each well to evaluate cell viability. A color shift from blue/purple (absence of metabolic activity/dead cells) to pink (presence of metabolic activity/viable cells) was used to visually determine microbial growth [53]. The MIC was defined as the lowest concentration of FuCho that completely inhibited visible growth. All assays were performed in triplicate (*n* = 3).

### 3.8. Effect of FuCho on Bacterial Morphology, Cell Viability and Membrane Integrity Assay

Microbial samples were prepared for confocal laser scanning microscopy (CLSM) to evaluate cell viability and membrane integrity. Suspensions of *Staphylococcus aureus* (ATCC 6538) and *Escherichia coli* (ATCC 25922) were prepared in Brain Heart Infusion (BHI) broth (1.0 mL; approx. $1.5\times 10^{8}\;\mathrm{CFU}/\mathrm{mL}$) and either left untreated (negative control), exposed to FuCho obtained from *C. elegans* or *A. caatinguensis* at their respective minimum inhibitory concentrations (MIC), or treated with 70% (*v*/*v*) isopropyl alcohol (positive control for cell death) for 30 min at 37 °C.

Following treatment, cells were harvested by centrifugation (4000× *g*, 10 min), resuspended in phosphate-buffered saline (PBS, pH 7.4), and stained using the LIVE/DEAD™ BacLight™ Bacterial Viability Kit (Invitrogen, Thermo Fisher Scientific, São Paulo, Brazil) according to the manufacturer’s instructions [54]. The staining solution contained SYTO 9 (3.34 μM in DMSO), a green fluorescent nucleic acid stain that freely penetrates intact cell membranes, and propidium iodide (PI, 20 μM in DMSO), a red fluorescent dye that enters only cells with compromised plasma membranes. Dyes were incubated simultaneously in PBS for 15 min at 25 °C in the dark.

Confocal images were acquired using a Leica TCS SP2/AOBS confocal laser scanning microscope (Leica Microsystems, Mannheim, Germany). SYTO 9 fluorescence was excited at $\lambda_{\mathrm{ex}}=488\;\mathrm{nm}$ and detected at $\lambda_{\mathrm{em}}=500\text{–}540\;\mathrm{nm}$, whereas PI fluorescence was excited at $\lambda_{\mathrm{ex}}=543\;\mathrm{nm}$ and detected at $\lambda_{\mathrm{em}}=610\text{–}650\;\mathrm{nm}$ [55,56]. Image analysis and colocalization scatterplots were generated to evaluate fluorophore distribution and pixel intensity profiles.

### 3.9. Statistical Analysis

Experimental data are presented as the mean ± standard deviation (SD) of independent replicates (*n* = 3). Statistical analyses were performed using STATISTICA software (version 7.0, StatSoft Inc., Tulsa, OK, USA). Descriptive statistics were followed by inferential analysis using one-way analysis of variance (ANOVA) combined with Tukey’s post hoc test to evaluate significant differences among groups (p<0.05).

For the $2^{2}$ central composite rotatable design (CCRD), response surface methodology (RSM) and three-dimensional surface plots (or two-dimensional contour plots) were generated to evaluate the main and interaction effects of the independent variables on the responses. A second-order polynomial regression model was fitted to the experimental data, and term significance was established at p<0.05. Model adequacy and goodness-of-fit were assessed using the coefficient of determination ($R^{2}$), Fisher’s ratio (F-value), statistical significance (p-value), and adequate precision [40].

## 4. Conclusions

The sustainable substrate CSL-GLY demonstrated potential as an alternative culture medium for fungal chitosan (FuCho) production by Mucorales fungi. Among the nine tested strains, *Absidia caatinguensis* URM 7156 and *Cunninghamella elegans* UCP 1306, isolated from the Caatinga biome, showed the highest chitosan production in CSL-GLY media. The concentrations of these substrates in cultivation media influenced chitosan production, with the highest specific yield obtained in medium containing 4% CSL, 2% GLY for *Absidia caatinguensis* URM 7156, and 1.18% CSL, 2% GLY for *Cunninghamella elegans* UCP 1306. To the best of our knowledge, this is the first study to report CSL-GLY mixtures as a nutrient source for FuCho production by Mucorales fungi. Furthermore, FuCho showed inhibitory effects against pathogenic species of bacteria and *Candida,* causing cell injury and membrane-compromise in *E.coli* and *S. aureus*, while presenting no irritation potential to the mucosa of mammals in in vitro tests.

## Figures and Tables

**Table 1:** Yields of biomass (g/L), and chitosan (FuCho; mg/g) obtained by fungal strains (order Mucorales) grown in a culture medium based on corn steep liquor (CSL; 4% *v*/*v*) and glycerol (GLY; 2%, *v*/*v*).

Fungal Strains	Biomass (g/L)	FuCho (mg/g)
*Absidia caatinguensis* URM 7156	7.94 (±1.63) ^abc^	126.56 (±1.25) ^a^
*Cunninghamella elegans* UCP 1306	9.03 (±1.02) ^ab^	92.99 (±0.87) ^ab^
*Backusella constricta* URM 7323	8.47 (±1.61) ^abc^	63.41 (±3.08) ^abc^
*Syncephalastrum racemosum* UCP 1302	10.36 (±1.01) ^a^	60.10 (±1.68) ^abc^
*Cunninghamella phaeospora* UCP 1303	9.25 (±1.84) ^ab^	50.63 (±6.65) ^abc^
*Mucor pernambucoensis* URM7640	6.99 (±0.37) ^bcd^	44.70 (±2.96) ^bc^
*Rhizopus arrhizus* UCP 1295	10.61 (±1.49) ^a^	37.08 (±1.52) ^bc^
*Lichtheimia brasiliensis* URM 6910	5.89 (±0.31) ^cd^	37.07 (±3.69) ^bc^
*Absidia aguabelensis* URM 8213	4.17 (±0.49) ^d^	17.88 (±0.88) ^c^

Values represent the mean ± standard deviation (SD) of four independent replicates (*n* = 4). Means in the same column followed by different superscript letters are significantly different (*p* < 0.05) based on Tukey’s test.

**Table 2:** Biomass (g/L) and chitosan (FuCho; mg/g) produced by *Absidia caatinguensis* URM 7156 and *Cunninghamella elegans* UCP 1306 in each experimental run with different concentrations of corn steep liquor (CSL; %, *v*/*v*) and glycerol (GLY; %, *v*/*v*) using a $2^{2}$ central composite rotatable design (CCRD).

Run	CSL (%)	GLY (%)	*Absidia caatinguensis*		*Cunninghamella elegans*	
			Biomass (g/L)	FuCho (mg/g)	Biomass (g/L)	FuCho (mg/g)
1	2	1	7.76	48.62	5.63	86.39
2	2	3	9.57	56.69	6.07	38.89
3	6	1	4.79	34.59	10.88	105.74
4	6	3	12.20	64.68	15.27	76.4
5	1.18	2	7.08	29.02	5.21	164.92
6	6.82	2	7.68	40.65	13.98	66.37
7	4	0.59	5.44	35.65	7.48	89.63
8	4	3.41	11.34	71.44	11.01	50.33
9	4	2	10.07	128.26	10.52	83.77
10	4	2	10.26	114.77	8.68	76.84
11	4	2	11.64	111.71	9.16	82.24
12	4	2	10.66	124.77	10.43	99.04

**Table 3:** Yields of biomass (g/L), and fungal chitosan (FuCho; mg/g), including its deacetylation degree (DD%), obtained by various fungal strains under specific culture conditions compared with *Absidia caatinguensis* URM 7156 and *Cunninghamella elegans* UCP 1306 in the present study.

Fungal Strains	Culture Conditions	Biomass (g/L)	FuCho (mg/g)	DD%	Reference
*Cunninghamella elegans* UCP 1306	1.18% CSL, 2% GLY, 28 °C, 150 rpm, 96 h	5.21	164.92 (16.49%)	75	This study
*Absidia caatinguensis* URM 7156	4% CSL, 2% GLY, 28 °C, 150 rpm, 96 h	10.07	128.26 (12.83%)	80	This study
*Rhizopus arrhizus* UCP 402	6% CSL and 13.24% Honey, 28 °C, 150 rpm, 96 h	11.71	29.3	86	[9]
*Lichtheimia* *hyalospora* UCP 1266	4%CSL, 6% CWW, 28 °C, 150 rpm, 120 h	6.298	44.91	83.61	[10]
*Cunninghamella elegans* UCP 0542	CSL 5.0% (*v*/*v*) and molasses 2.5% (*v*/*v*), 28 °C, 150 rpm, 96 h	13.58	33.13	80	[13]
*Rhizopus arrhizus* UCP 0402	CSL 2.0% (*v*/*v*) and molasses 1.0% (*v*/*v*), 28 °C, 150 rpm, 96 h	8.25	49.31	82	[13]
*Mucor racemosus* IFM 40781	YPD medium (0.3% yeast extract, 1% polypeptone and 2% D-Glucose pH 4.5, 100 rpm, 28 °C, 96 h	15.0	35.1	-	[14]
*Cunninghamella elegans* URM 46109	YPD medium (0.3% yeast extract, 1% polypeptone and 2% D-Glucose pH 4.5, 100 rpm, 28 °C, 96 h	25.0	20.5	-	[14]
*Cunninghamella elegans* UCP 1306	40% CAJ, 30% CW, 28 °C, 150 rpm, 96 h	12.21	64.09	75	[15]
*Cunninghamella elegans*	YPD 10 g/L, peptone 20 g/L, dextrose 20 g/L, 28 °C, 96 h in static mode	-	20.69	76	[16]
*Cunninghamella elegans* CBMAI 0843	YPD medium, room temperature, 120 h, 100 rpm	10.78	1.89%	-	[18]
*Fusarium verticillioides* FV01/07	3 g Arginine, 1.4 g NH_4_Cl, 1.5 g K_2_HPO_4_, 1.4 g FeSO_4_, 0.1 g NaCl, 180 rpm, 3 week	-	72.26	82.43	[27]
*Rhizopus stolonifer*	PDA, 28 °C, 100 rpm, 72 h	5.48	40.59	85.44	[28]
*Rhizopus oryzae* NRRL 1526	PDB, 30 °C, 180 rpm, 6 days	8.10	18.6%	68.34	[29]
*Rhizopus oryzae*	5% moisture, 50% rice husk, and 1.8 g/L Urea, 30 °C, 48 h	-	31.5%	86.4 ± 0.6	[30]
*Cunninghamella elegans* UCP 1306	2% CSL, 10% CWW, 35 °C, 150 rpm, 96 h	3.58	132.42	80.7	[31]

**Table 4:** Minimum inhibitory concentration (MIC) of fungal chitosan (FuCho) extracted from the biomass of *Absidia caatinguensis* and *Cunninghamella elegans* against pathogenic microorganisms. Chlorhexidine was used as a positive control.

Microorganism	Test Substances (mg/mL)		
	FuCho *C. elegans*	FuCho *A. caatinguensis*	Chlorhexidine
*Staphylococcus aureus*	3.0	2.0	0.48
*Staphylococcus epidermidis*	2.0	2.0	0.48
*Enterococcus faecium*	5.0	4.0	0.6
*Streptococcus gordonii*	2.0	1.0	0.48
*Streptococcus oralis*	1.0	1.0	0.48
*Streptococcus mutans*	1.0	1.0	0.48
*Streptococcus salivarius*	1.5	1.0	0.48
*Pseudomonas aeruginosa*	2.0	2.0	0.6
*Escherichia coli*	2.0	2.0	0.6
*Candida* *krusei*	4.0	3.0	0.24
*Candida albicans*	4.0	3.0	0.24

## Data Availability

The original contributions presented in this study are included in the article. Further inquiries can be directed to the corresponding author.
